# Chemistry and Pharmacology of the Kazakh *Crataegus Almaatensis* Pojark: An Asian Herbal Medicine

**DOI:** 10.3390/antiox8080300

**Published:** 2019-08-10

**Authors:** Sabrina S. Soares, Elmira Bekbolatova, Maria Dulce Cotrim, Zuriyadda Sakipova, Liliya Ibragimova, Wirginia Kukula-Koch, Thais B. Sardella Giorno, Patrícia D. Fernandes, Diogo André Fonseca, Fabio Boylan

**Affiliations:** 1 Laboratory of Pharmacy and Pharmaceutical care, Faculty of Pharmacy, University of Coimbra, 3000-548 Coimbra, Portugal; 2 School of Pharmacy and Pharmaceutical Sciences & Trinity Biomedical Sciences Institute, Trinity College Dublin, Dublin 2 D02 PN40, Ireland; 3 School of Pharmacy, JSC National Medical University, 050000 Almaty, Kazakhstan; 4 Coimbra Institute for Clinical and Biomedical Research (iCBR), Faculty of Medicine, University of Coimbra, 3000-548 Coimbra, Portugal; 5 CIBB Center for Innovative Biomedicine and Biotechnology, University of Coimbra, 3000-548 Coimbra, Portugal; 6 Department of Pharmacognosy with Medicinal Plants Unit, Medical University of Lublin, 1 Chodzki str., 20-093 Lublin, Poland; 7 Laboratório da Dor e Inflamação, Universidade Federal do Rio de Janeiro, 21941-902 Rio de Janeiro, Brazil

**Keywords:** *Crataegus* almaatensis Pojark, Kazakhstan, HSCCC, Flavonoids, vascular activity, anti-inflammatory activity

## Abstract

*Crataegus almaatensis*, an endemic ornamental plant in Kazakhstan is used in popular medicine due to its cardiotonic properties. The most studied species of the same genus are commonly found in Europe, which shows the importance of having the Kazakh species validated via its chemical and pharmacological studies. High-speed countercurrent chromatography (HSCCC) operated under optimized conditions enabled an isolation of the three main compounds from the aqueous phase of the leaves ethanol extract, further identified by nuclear magnetic resonance (NMR), as quercetin 3-O-rhamnoside (quercitrin) (4.02% of the crude extract-CECa); quercetin 3-O-β-galactoside (hyperoside) (1.82% of CECa); kaempferol 3-O-α-L-rhamnoside (afzelin) (0.94% of CECa). The CECa, the aqueous phase of the crude extract (APCa) together with the isolates were evaluated for their vascular (vascular reactivity in human internal mammary artery-HIMA), anti-nociceptive (formalin-induced liking response and hot plate) and anti-inflammatory (subcutaneous air-pouch model-SAP) activities. CECa at the concentrations of 0.014 and 0.14 mg/mL significantly increased the maximum contractility response of HIMA to noradrenaline. The APCa CR curve (0.007–0.7 mg/mL) showed an intrinsic relaxation effect of the HIMA. APCa at the dose of 100 mg/kg i.p. significantly decreased the total leukocyte count and the IL-1β release in the SAP wash.

## 1. Introduction

The genus *Crataegus* spp., subfamily Maloideae, family Rosaceae, commonly known as Hawthorn, is native to temperate zones and commonly found in Europe, Asia and North America [1]. *Crataegus* is represented by seven species in the Kazakh flora, from which only *C. almaatensis* is known to be endogenous. It is a dense, spiny tree up to 3–4 m tall, with cherry colored branches, thorns are rare 1–2 cm long, leaves elliptical-avoid, sharp, but laciniate in the bottom. Berries are dark purple, juicy, with reddish pulp and up to 3–5 seeds. Trees produce dense white colored flowers. It is spread in the foothills of Ile-Alatau Mountains, Kyrgys alatau and Karatau [2,3]. More than 20 species of *Crataegus* are currently consumed as medicines or as a basis for the production of drugs. Extracts of *C. laevigata* and *C. monogyna* are widely used in the treatment of cardiovascular diseases throughout Europe due to their efficacy and safety; these species are listed in the pharmacopoeia of several countries, including Germany, France, and England [4]. In Kazakhstan the species *Crataegus almaatensis* Pojark (*C. almaatensis*), is deployed in popular medicine due to its cardiotonic properties [5]. In relation to the cardiovascular activity, products based on *Crataegus* spp. are currently used as an alternative in the treatment of hypertension, angina, arrhythmia, and mild congestive heart disease [1]. *Crataegus* spp. are also used for digestive and endocrine system ailments [6]. These species have also demonstrated anti-ischemic [7], anti-inflammatory [8], anti-microbial, gastroprotective, free radical scavenging [9,10], and anti-fungal effects [11]. They have positive effect on glucose and lipid metabolism regulation, thus being also recommended as an anti-hyperglycemic and anti-hyperlipidemic agent [12].

The pharmacological activities of the *Crataegus* species are attributed mainly to polyphenolic compounds of the genus. In general, flavonol- and flavanone-type flavonoids are present in flowers and leaves at a higher level, while proanthocyanidins are abundant in berries [13]. There are a few scientific papers on the biochemical studies of cultivated *C. almaatensis* fruits, on its determination of carotene, bioflavonoids, sugars and organic acids content carried out by Russian researchers [14,15]. In their previous findings, the authors of this manuscript denoted a marked antioxidant potential of various extracts of the plant, which were correlated with the presence of phenolic components tentatively identified in the LC-MS analyses [16]. However, separation of single major components from hawthorn has been of great interest lately [17], as it can assist further pharmaceutical and pharmacological studies.

When comparing to well-known conventional methods, high speed counter current chromatography (HSCCC) is a liquid-liquid based chromatography that allows the separation of substances in a short time, with the use of a great variety of solvents, and as a support free partition technique. It eliminates the drawback of irreversible adsorption of the sample to the solid support and thus allowing full recovery of the sample [18].

This study aimed at the isolation of the main constituents of *C. almaatensis* leaves ethanol extract by means of HSCCC. Following isolation, nuclear magnetic resonance (NMR) and high-performance liquid chromatography (HPLC) were used to promote the identification and quantification of the isolated components, respectively. The therapeutic profile of *C. almaatensis* was evaluated by means of different models—in vitro for vasoactivity, and in vivo for nociception and inflammation.

## 2. Materials and Methods 

The phytochemical profile in the present study was established combining HSCCC with different techniques of purification, NMR and HPLC.

### 2.1. Reagents and Materials

The solvents hexane, ethyl acetate, methanol and acetic acid were purchased from Trinity College Dublin HMF facilities. HPLC grade methanol and orthophosphoric acid were purchased from Sigma-Aldrich, Ireland. HPLC grade water was obtained from a deionized water treatment system from PureLab Option. All spectra were measured at 25 °C in deuterated methanol (MeOD) using Tetramethylsilane (TMS) as an internal standard. Noradrenaline, formalin and carrageenan were purchased from Sigma-Aldrich, Dublin, Ireland.

### 2.2. Plant Material

The extract investigated in this study was obtained from the leaves of *Crataegus almaatensis* Pojark. The plant was collected in the Almaty region, Kazakhstan in May 2015 and authenticated by the Institute of Botany and Phytointroduction. A specimen was deposited under the accession number 01–04/456 dated to 10.11.2016. One hundred grams of ground plant material was extracted in a Soxhlet apparatus with absolute ethanol for 36 h. The ethanol extract was evaporated on rotary evaporator (IKA HB10 control attached to Fisher Scientific pump) and suspended in the solvent system used for HSCCC separation.

### 2.3. HSCCC Separation Procedure

The HSCCC was performed using an instrument IntroPrepTM (Quattro). The apparatus works by the action of centrifuge force in a rotation speed of 200 g. The column is a coil of PTFE tubing wrapped around a bobbin (diameter of the tube 2.0 mm, total volume 136 mL) and a 5 mL manual sample loop. Four solvent systems (SS) composed of HEMWat (Hexane: ethyl acetate: methanol: water) at the proportions of 1:1:1:1; 1:2:1:2; 1:3:1:3 and 1:4:1:4 were tested to choose the best separation for both the crude ethanol extract and its aqueous phase. The solvent system (SS) utilized for this study was chosen according to the partitioning of the constituents of the plant material in both the upper and lower phases of the biphasic solvent system. Both crude extract (CECa) and aqueous phase (APCa) together with the isolated compounds were used to evaluate the pharmacological activities.

### 2.4. Purification of Isolated Compounds

After successive cycles of HSCCC, the collected samples were grouped into five fractions according to the similarity of elution pattern and retention factor (Rf) observed in the TLC plates. Three semi-purified fractions were submitted to Gel-filtration Chromatography using a column Sephadex LH-20 obtained from Sigma-Aldrich (Dublin, Ireland), eluted with methanol at an average flow rate of 0.5 mL/min. A complete purification was not achieved for one of these fractions with Sephadex LH-20 gel filtration and preparative TLC plates (PTLC) were used as the last purification step. The fraction was spotted multiple times on a silica gel 20 × 20 cm PTLC UNIPLATE, obtained from Analtech-USA (New Jersey) and eluted with ethyl acetate: acetic acid: water in a ratio of 3:1:1. The pure compound was then scrapped off the plate and then dissolved in methanol for further analysis.

### 2.5. NMR Identification

Structural elucidation of the isolated molecules was performed using an Agilent Technology (USA) 400NMR apparatus. Hydrogen and carbon 13 spectra were recorded on a BRUKER TOPSPIN 2.1 (1H-NMR: 400 and 600 MHz and 13C-NMR: 125 MHz) NMR spectrometry system using the Bruker pulse sequence standard. Two-dimensional measurements (H-H COSY, HMBC, HMQC) were obtained on the same instrument with the usual pulse sequences. Peaks were visualized from ACD/NMR Processor Academic Edition software. 

### 2.6. HPLC Quantification

HPLC system (Waters) was used in conjunction with Waters 1525 Binary HPLC pump, Waters 2487 dual λ absorbance detector, Waters 717 plus auto sampler and Breeze software program. Reversed phase C-18 Kromasil (250 × 4.6 mm) column was used. A stock solution was prepared using 7.5 mg/mL of each purified flavonoid. They were then diluted to the following concentrations: 1250 µg/mL, 625 µg/mL, 312.5 µg/mL, 156.25 µg/mL, 79.12 µg/mL, 39.06 µg/mL and 19.53 µg/mL. The sample solution used here was the crude extract in a 1250 µg/mL concentration. All samples were analyzed in triplicate and a calibration curve was constructed. The mobile phase consisted of (A) 0.25% orthophosphoric acid in water and (B) HPLC graded methanol and run in a gradient mode 40% B for 5 min, 55% B for 5 to 10 min, 65% B for 10 to 15 min, 50% B for 15 to 20 min and 30% B for 25 to 30 min. The flow rate was 1 mL/min and the injection volume 5µL. Spectra were recorded at 254 and 280 nm (at 25 degrees Celsius).

### 2.7. Pharmacological Activity

After separation of the compounds in a sufficient amount, the pharmacological activity was investigated together with the CECa and APCa in different in vitro and in vivo models.

### 2.8. Vascular Reactivity Model in Vitro: Human Internal Mammary Artery (HIMA)

Human internal mammary arteries (HIMAs) were collected from patients undergoing coronary bypass surgery at the Cardiothoracic Surgery Service of the University Hospital of Coimbra (Portugal) with the approval of the local research ethics committee (PC-388/08). After harvesting, the arterial samples were isolated by removal of the adjacent connective tissue. The arteries were cut into 3mm-long rings, suspended on stainless steel hooks under a passive force of 19.6 mN in 10mL organ baths (Panlab, Barcelona, Spain) filled with a Krebs-Henseleit solution aerated with 5% CO_2_–95% O_2_ and maintained at 37 °C. After an equilibration period of 2 h, with time from time washings of the preparations, cumulative concentration-response (CR) curves for the extracts (0.007–0.7 mg/mL) were recorded using a Panlab isometric transducer (Barcelona, Spain) connected to a PowerLab data acquisition package (ADInstruments, Sydney, Australia). Incubation of the rings with the extracts (0.014, 0.028 and 0.14 mg/mL) in between the two CR curves to noradrenaline was performed. The effect of the extracts on HIMA relaxation after a pre-contraction to noradrenaline (20 μM) was also evaluated. After the equilibration period and at the end of each assay, a single application of 60 mM KCl before the first CR curve was used to challenge and to confirm the sustained functional viability of the organ. The rings that did not produce stable contractile responses to KCl over the course of the experiment were not included in the final data in accordance with Silva et al. [19].

### 2.9. Animals

Male and female Swiss Webster mice (25–40 g) kept in a controlled temperature environment (22 ± 2 °C, 60–80% humidity) with food and water ad libitum regimen for 24 h were used in all in vivo experiments (groups of 5–7 animals). The protocol for the animal experiments was approved by the National Council for Control of Animal Experimentation (CONCEA), Biomedical Science Institute/UFRJ and Ethical Committee for Animal Research, under the number DFBCICB015–04/16.

### 2.10. Anti-Nociceptive and Anti-Inflammatory Activity In Vivo

Stock solutions of 100 mg/mL of the dried extracts, isolated compounds and positive control (ASA and Morphine) were prepared by dissolving in DMSO and kept at −20 °C.

Formalin-induced liking response. 

Acute pain was induced by an injection of 20 µL of formalin (2.5% *v/v*) into the dorsal surface of the left hind paw according the method described by Giorno et al., [20]. The time of licking was recorded during two phases: the first phase (neurogenic pain response) from immediately to 5 min after injection and the second phase (inflammatory pain response) between 15 to 30 min post-injection. The animals were pre-treated with intraperitoneal doses of the extracts (10, 30 and 100 mg/Kg), isolated compounds (0.3, 1 and 3 mg/Kg), ASA (10 mg/Kg), morphine (2.5 mg/Kg) or vehicle (ultrapure water) 30 min before formalin injection.

Hot Plate. 

Mice were tested using the method described by Sobrinho et al., [21]. Animals were placed on a hot plate (Insight Equipment, São Paulo, Brazil) set at 55 ± 1 °C. The reaction time was recorded (jumping, licking or lifting the back paw) 30, 60, 90, 120, 150 and 180 min after intraperitoneal administration of the extracts (10, 30 and 100 mg/Kg), isolated compounds (0.3, 1, and 3 mg/Kg), morphine (2.5 mg/Kg) or vehicle (ultrapure water). Baseline was considered the mean of the reaction time 60 and 30 min before treatments and it was defined as the normal reaction of the animal to the temperature. Anti-nociception was quantified as the increase in the baseline (%) calculated by the formula: (reaction time × 100/baseline – 100).

Subcutaneous air-pouch (SAP) model. 

The air pouches were confectioned and maintained by two consecutive administrations of sterile air (8 and 10 mL, respectively) in the intraescapular area of the mice with intervals of four days. Two days after the last injection, animals received sterile carrageenan suspension (1% 1 mL) or sterile saline (1mL) in the SAP. Mice were pre-treated intraperitoneally with 100 mg/Kg of the extracts or ASA 10 mg/Kg 30 min before the carrageenan administration. Animals were sacrificed 24 h after carrageenan injection, and the cavity was washed with 1ml of sterile saline. Aliquots of the exudates together with a blood sample and a bone narrow wash (1 mL sterile saline in femur) were collected for determination of total number of cells. The exudates were centrifuged at 1200 rpm for 10 min at 4 °C, and the supernatants were collected and stored at –20 °C until use based on the work by Raymundo et al. [22].

IL-1β, TNF-α, IFN-γ, and protein measurements. 

Supernatants from the exudates collected in the SAP were used to determine Interleukin-1β (IL-1β), Tumour Necrosis Factor-α (TNF-α), Interferon-γ (IFN-γ), and protein measurements according to Raymundo et al. [22]. The cytokines were determined by enzyme-linked immunosorbent assay (ELISA) according the protocol supplied by the manufacturer (BD Biosciences). The protein content of each supernatant was determined using the BCA method (BCATM, Thermo Fisher Scientific, Inc, Dublin, Ireland).

Nitrate measurement. 

The nitric oxide (NO) production was evaluated by measuring the concentration of nitrate in the exudates of the SAP using the nitrate conversion protocol described in Bartholomew [23], modified by Raymundo et al. [22], followed by the Griess reaction described in Green et al. [24]. An equal volume of the Griess reagent (1 mL of 1:1 0.1% of naphthyl-ethylenediamine and 1% sulfanilamide in 5% phosphoric acid) was mixed with exudate. The absorbance was determined using a microplate reader at 540 nm.

### 2.11. Statistical Analysis

In vivo experimental groups were composed of 5–7 animals. Results are expressed as the mean ± standard deviation (S.D.) for the formalin-induced liking response, subcutaneous air-pouch model. The hot plate results were quantified as area under curve, expressed as mean ± standard error (SEM) compared to control. Cardiovascular in vitro experiment was quantified as mean ± SEM. Statistical significance was calculated by analysis of variance (ANOVA) followed by Bonferroni post-test. P values less than 0.05 (*p < 0.05) and 0.01 (**p < 0.01) were considered significant.

## 3. Results

### 3.1. Phytochemistry

HEMWat (Hexane: ethyl acetate: methanol: water) in a ratio of 1:4:1:4 presented the best distribution of the compounds of interest in the lower or aqueous phase and upper or organic phase when analysed on a TLC plate using ethyl acetate: acetic acid: water in a ratio of 6:1:1, as mobile phase. Initially 80 tubes of the ethanol extract from leaves of *Crataegus almaatensis* were collected from the HSCCC using HEMWat as a solvent system as mentioned before. The tubes were joined into fractions accordingly the chromatographic similarities of the constituents (Rf value observed on the TLC plates). Compound 1 (Rf~0.46) and compound 2 (Rf~0.81) were isolated and purified by means of gel filtration chromatography and PTLC. Aiming at an optimization of the isolation, only the aqueous phase of the leaves extract, after drying and diluting it in HEMWat, was used for the following HSCCC runs. The two previously mentioned compounds were again isolated and purified only by recrystallization with methanol. A third compound (Rf~0.88), was also isolated and purified by gel filtration chromatography and PTLC. 

#### 3.1.1. Structure Elucidation

**Figure 1:**
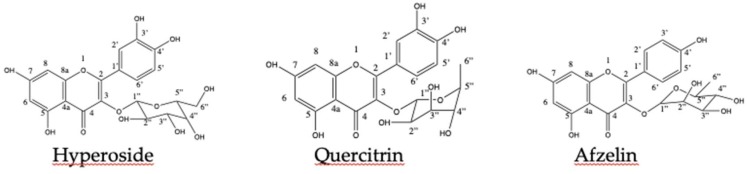
Chemical compounds isolated from aqueous phase of the crude extract (APca).

The structure elucidation of the isolated compounds was performed by means 1H NMR and 13C NMR and compared to the literature. According to the data from literature the compounds were identified as Quercetin 3-O-β-galactoside (Hyperoside), Quercetin-3-O-rhamnoside (Quercitrin), and Kaempferol 3-O-α-l-rhamnoside (Afzelin), respectively (Figure 1). The chemical signals obtained for the isolated substances are described (in ppm) as follows:

Compound 1: yellow powder: Quercetin 3-O-β-galactoside (Hyperoside) (88.5 mg); ^1^H NMR (MeOD, 400 MHz) δ ppm: 12.60 (1H, *s*, OH-5); 10.83-9.11(3H, *s*, OH), 7.62 (1H, *dd*, J = 8Hz/2Hz, H-6′); 7.50 (1H, *d*, J = 2 Hz, H-2′); 6.78 (1H, *d*, J = 8 Hz, H-5′); 6.37 (1H, *d*, J = 2 Hz, H-8); 6.17 (1H, *d*, J = 2 Hz, H-6); 5.34 (1H, *d*, J = 8 Hz, H-1”); 5.09-3.14 (4H, sugar). ^13^C NMR (125 MHz, MeOD): 177.95 (C-4); 164.60 (C-7); 161.70 (C-5); 156.68 (C-2); 156.68 (C-8a); 148.89 (C-4′); 145.30 (C-3′); 133.92 (C-3); 122.49 (C-6′); 121.55 (C-1′); 116.38 (C-5′); 115.65 (C-2′); 104.37 (C-4a); 102.21 (C1”); 99.31 (C-6); 93.97 (C-8); 76.31 (C-5”); 73.63 (C- 3”), 71.66 (C-2”); 68.38 (C-4”); 60.59 (C-6”).

Compound 2: yellow powder: Quercetin 3-O-rhamnoside (Quercitrin): (209 mg); ^1^HNMR (MeOD, 400 MHz) δ ppm: 12.67 (1H, s, OH-5); 10.83-9.31(3H, s, OH), 7.27 (1H, *s*, J = 2 Hz, H-2′); 7.22 (1H, *dd*, J = 8 Hz/2 Hz, H-6′); 6.84 (1H, *d*, J = 8 Hz, H-5′); 6.36 (1H, *d*, J = 2 Hz, H-8); 6.18 (1H, *d*, J = 2 Hz, H-6); 5.23 (1H, *d*, J = 1.5 Hz, H-1”); 4.92-3.09 (4H, sugar); 0.79 (3H, *d*, J = 6 Hz, H-6”). ^13^C NMR (125 MHz, MeOD): 178.22 (C-4); 164.65 (C-7); 161.76 (C-5); 157.78 (C-2), 156.91 (C-8a); 148.89 (C-4′); 145.66 (C-3′); 134.66 (C-3); 121.58 (C-6′); 121.19 (C- 1′); 116.11 15(C-5′); 115.93 (C-2′); 104.54 (C-4a); 102.28 (C-1”);99.15 (C-6); 94.09 (C-8); 71.62 (C-5”); 71.06 (C- 3”); 70.80 (C-4”); 70.52 (C-2”); 17.97 (C-6”).

Compound 3: yellow crystal: kaempferol 3-O-α-L-rhamnoside (Afzelin): (37.2 mg); 1H NMR (MeOD, 400 MHz) δ: 7.74 (2H, *d*, J = 8 Hz, H2′, H6′); 6.92 (2H, *d*, J = 8 Hz, H3′, H5′); 6.33 (1H, *d*, J = 2 Hz, H-8); 6.17 (1H, *d*, J = 2Hz, H-6); 5.37 (2H, *s*, J = 2 Hz, H-1”); 4.5-3 (4H, sugar); 0.91 (3H, *d*, J = 8 Hz, H-6”). 

#### 3.1.2. HPLC Quantification

**Figure 2:**
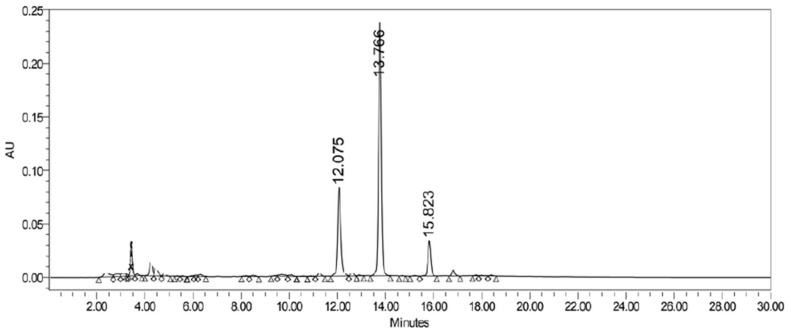
High-performance liquid chromatography (HPLC) of crude extract-CECa (Ceca) showing the peaks for Hyperoside (12.08 min), Quercitrin (13.77 min) and Afzelin (15.82 min).

The HPLC analysis showed a retention time of approximately 15.2 min for afzelin, 13.4 min for quercitrin, and 11.7 min for hyperoside (Figure 2). The calibration curves were based on the method described and validated by Sakipova et al. [25] and all substances showed good linearity with regression coefficients R^2^ ≥ 0.99 [26]. The calibration curves were used to quantify the substances in the total extract using the CECa chromatogram at a concentration of 1250 µg/mL. The concentrations of 9.4 mg of afzelin/g of CECa, 40.02 mg of quercitrin/g of CECa and 18.2 mg of hyperoside/g of CECa were obtained, representing a total of 0.94%, 4.02% and 1.82%, respectively.

### 3.2. Pharmacology

#### 3.2.1. Vascular Activity of *C. almaatensis* Extracts

**Figure 3:**
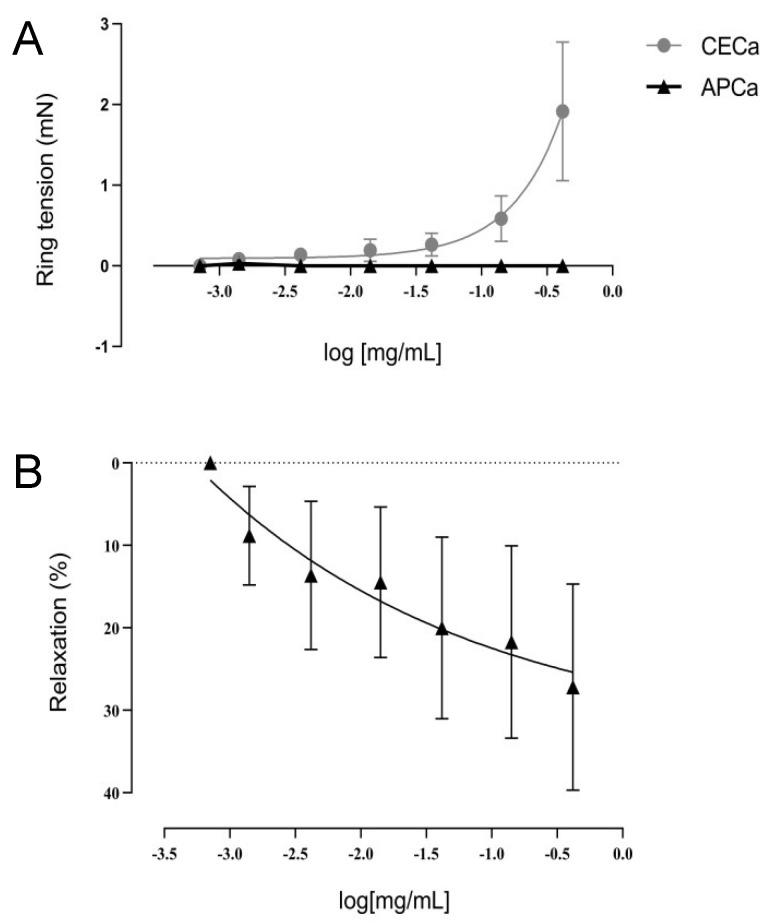
Concentration-response curves to the extracts CECa and APCa from 0.007 to 0.7 mg/mL (**A**) and relaxation curve of the APCa at the concentrations from 0.07 to 7 mg/mL after a pre-contraction to noradrenaline 20 µM (**B**). Results are presented in mean ± SEM.

No contractile effect was observed when the HIMA rings were submitted to a CR curve to APCa at the tested concentrations. However, a contractile effect was observed for the CECa. A relaxation effect of the HIMA was evident when the rings were submitted to a pre-contraction with noradrenaline 20µM followed by the administration of cumulative concentrations of the APCa (0.07–7mg/mL) into the organ baths (Figure 3). When the artery rings were incubated with different concentrations of the extracts (0.014, 0.28, 0.14 mg/mL) into the organ baths, CECa demonstrated an increase in the %Emax of the CR curve to noradrenaline. The incubation and of the HIMA with APCa did not demonstrate a significant effect on the %Emax of the CR curve to noradrenaline (Table 1). 

#### 3.2.2. Anti-Nociceptive and Anti-Inflammatory Activity of *C. almaatensis*

**Figure 4:**
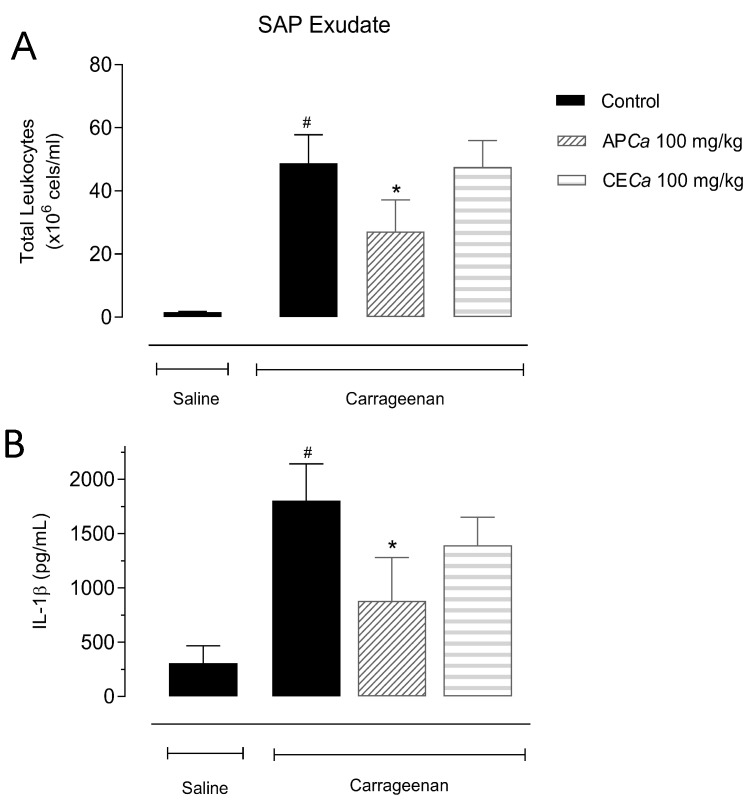
Effects of CECa and APCa on total leukocytes counts (**A**) and IL-1β (**B**) in the SAP exudate in the subcutaneous air-pouch model. Animals were treated with CECa (100 mg/Kg i.p.) and APCa (100 mg/Kg i.p.) 30 min prior the carrageenan injection in the SAP. Results are presented in mean ± SD (*p < 0.05).’*p < 0.05 compared to the treated group. #*p* > 0.05 compared to the saline-control group.

The results obtained do not demonstrate any anti-nociceptive effect of the *C. almaatensis* leaves ethanol extract or its isolated compounds in the doses administered when compared to control in the hot plate and formalin-induced liking response tests (results not shown). When 1 mL of carrageenan (1% in saline) was injected into the mice air pouch it was observed a significantly increased in total leukocyte and protein levels in the exudate compared to the control saline. Additionally, carrageenan raised the levels of inflammatory mediators TNFα, IFN-γ, IL-1β and nitric oxide concentrations in the exudate when compared to control saline. However, no significant effect was observed regarding the levels of protein and the other mediators in the exudate when mice were pre-treated with APCa and CECa (data not shown). Pre-treatment of mice with the APCa (100 mg/kg) significantly reduced the total leukocyte counts and suppressed IL-1β level in the exudate (Figure 4).

## 4. Discussion

The choice of the solvent system is one of the most important steps of the HSCCC. The HEMWat system described as being extremely versatile in the separation of constituents from complex mixtures was the SS of choice for this study. HEMWat has the ability to distribute from medium to high polar substances in their respective organic and aqueous phases [27]. The solvents belonging to this SS are the most used in the separation of natural products by HSCCC, probably due to their capacity to form a stable biphase, with a volume ratio of almost 1:1, and a high capacity for the separation of phenolic substances [28]. In addition, the presence of a glycosylated fraction gives the flavonoids an increase in polarity, and HEMWat is also effective at the highest polarities (between 1:2:1:2 and 1:6:1:6) as the combination applied in the present study [29]. Preliminary HPLC results on *C. almaatensis* quantification had shown concentrations in mg/g of 1.23 ± 0.05 and 19.79 ± 0.36 in the flowers, 51.00 ± 0.92 and 14.70 ± 0.37 in the leaves for compound 2 and 1, respectively. The concentration on the fruits was almost undetectable for compound 2 and 0.01 mg/g for compound 1. Based on the initial results and previous studies of the authors, further isolation of compounds was conducted from the ethanol extract using the leaves of *C. almaatensis* [16]. The attempt of optimizing the isolation technique applied initially, using only the aqueous phase of the leaves extract allowed the separation of the same two previously isolated compounds (hyperoside and quercitrin) in an almost purified state and the much less time-consuming method of recrystallization with methanol was able to completely purify them. The HSCCC optimisation also led to the isolation of a third compound (Rf~0.88) further purified by gel filtration chromatography and PTLC and further identified by 1H NMR as kaempferol 3-O-α-l-rhamnoside (Afzelin).

The identified compounds showed a retention time in the HPLC of approximately 11.7, 13.4 and 15.2 min for the hyperoside, quercitrin and afzelin, respectively. The retention time is inversely related to the level of hydroxylation and the higher the hydroxylation in a certain molecule, the bigger is its polarity, reducing the retention time, which is in accordance to our results [26]. The calibration curves enable for the quantification of the substances in the total extract using the CECa as previously mentioned. The concentrations of 18.2 mg/g of hyperoside, 40.02 mg/g of quercitrin and 9.4 mg/g of afzelin in the CECa were obtained, representing a total of 1.82%, 4.02% and 0.94%, respectively. Some Pharmacopoeias mention the minimum percentages of flavonoids accepted for different species of *Crataegus* genus. The Chinese Pharmacopoeia of 2005 refers an amount of not less than 7.0% for total flavonoids and 0.050% for hyperoside in leaves of the genus. The European (2004) and American Pharmacopoeia (1995 and 2011) accept a wider variety of species in a minimum amount of 1.5% of total flavonoids in the leaves [1]. Data from fifteen different species of *Crataegus* showed a maximum concentration of hyperoside of 3.06 mg/g in the fruits of *C. maximowiczii*, 3.08 mg/g in the flowers of *C. monogyna* and 9.53 mg/g in the leaves of *C. pinnatifida*. All concentations mentioned above are lower than the concentration of hyperoside obtained from the aqueous phase of *C. almaatensis* extract. The review does not mention quercitrin and afzelin as being major compounds in *Crataegus* spp. nor does it provide concentrations of these substances for comparison [1]. It is suggested from the data cited above that the evaluation in terms of the quality standard of the genus *Crataegus*, with respect to the composition of flavonoids, is based mainly on the number of total flavonoids. A total of 6.78% of flavonoids were isolated and identified from *C. almaatensis*, which is much higher than the minimum required by the quality standards of most of the pharmacopoeias consulted. The optimization technique applied in this study allowed for the isolation of bigger amounts of the identified substances in a shorter period of time, providing enough amounts for the identification, quantification and following pharmacological tests.

In terms of pharmacological actions, the APCa extract at concentrations of 0.007 to 0.7 mg/mL demonstrated inhibition of HIMA contraction following pre-contraction with a single administration of noradrenaline (20 µM). An assay using anesthetized rats in an induced myocardial ischemia model, different extracts of *C. meyeri* were administered, and a marked antiarrhythmic and anti-hypertensive effect was observed from the hydro-alcoholic extract, where the highest concentration of flavonoids, saponins and procyanidins were found [30]. Another study using procyanidin type B2, demonstrated intense endothelium-dependent HIMA rings vasodilation, demonstrating an increase in the synthesis and secretion of NO by endothelial cells and of prostacyclin, via the adenosine monophosphate (AMP) pathway, involving the activation of different K^+^ channels (which regulates contraction and vascular tone). In addition, the involvement of procyanidins in the modulation of intracellular Ca^2+^ release and Ca^2+^ uptake into the sarcoplasmic reticulum probably participates in NO-dependent HIMA relaxation [31,32]. Considering the high amount of procyanidins currently present in other species of *Crataegus* according to Edwards et al. [1], it is assumed that these substances are also present in the APCa extract and the HIMA rings relaxation effect observed is possibly related to the concentration of flavonoids and procyanidins in the extract. Procyanidins are compounds soluble in highly polar solvents, and a different solvent system selection would be necessary to achieve the separation of these compounds in the present assay. In addition, such compounds are usually isolated by chromatography in the form of flavan-3-ol-monomers or low molecular weight oligomers that make up the procyanidins [33]. When the CECa extract was administered to the artery bath at a range of concentrations from 0.014 to 0.14 mg/mL, a statistically significant increase was observed in maximal contraction to noradrenaline after 30 min incubation of the arterial rings. It is not possible to exclude the possibility of some other substance not isolated from the organic phase of the CECa extract, present in the total extract, but at the same time absent in APCa, to be responsible for the vasoconstrictor effect observed. A study using *Vitis vinifera* extract showed that the effect of procyanidins on vasodilation disappears in endothelium-denuded HIMA segments, demonstrating that the effect of procyanidins is dependent on the endothelium. However, the study did not observe an increase in maximal contraction to noradrenaline in the endothelium-denuded segments [34]. During manipulation of the segments in the assay, a damage of the endothelium may have occurred, but as described by Aldini et al. [34], this does not explain a significant increase in HIMA contraction. Novakovic et al. [31] demonstrated that the effect of procyanidins is concentration-dependent, being mediated by Ca^2+^ dependent potassium channels (K_Ca_), specifically intermediate conductance channels (IK_Ca_), and by calcium-carrying membrane proteins, specifically ATPase of Ca^2+^. The IK_Ca_ channels present in the vascular endothelium open in the presence of high concentrations of procyanidins producing relaxation, which do not happen at low concentrations of procyanidins. Ca^2+^-ATPase, however, is associated with Ca^2+^ reuptake to the sarcoplasmic reticulum, and only high concentrations of procyanidins can stop activating its reuptake activity. Considering the great variability in the concentrations of these substances in the different extracts and the fact that the CECa aqueous fraction would have a higher concentration of procyanidins due to its composition of polar solvents (methanol/water), it is possible to suppose a lower concentration of OPC’s in CECa. Consequently, a lower concentration of procyanidins in CECa could be related to the lack of effect on IK_Ca_ and Ca^2+^-ATPase, determining an increase in Ca^2+^ concentration, justifying the observed contraction. The currently most studied extracts of *C. almaatensis*, WS1442 (45% ethanolic extract) and LI132 (70% methanolic extract) are standardized dry extracts for 18.75% procyanidins and 2.2% flavonoids, respectively [32]. As procyanidins were not isolated in this work, its concentration in both extracts is unknown. Thus, the pathways involved in increasing the vascular tonus of HIMA by CECa could not be established, and for this reason, the mechanism of the increase in maximal contraction to noradrenaline by the crude extract requires further studies to be clarified.

In the present study, it was observed a lack of nociceptive effect for both extracts in the first phase of the formalin test and in the hot plate test. These results are in agreement with previous studies using the extracts of *Copaifera langsdorff* and *Balbisia calycina*, where afzelin, quercitrin and hyperoside were the major isolated compounds [35,36]. However, both studies had shown a significant effect of the extracts on the second phase of the pain induced by formalin test, suggesting a peripheral anti-inflammatory effect in the doses of 200 and 400 mg/Kg of *Copaifera langsdorff* and 800 mg/Kg of *Balbisia calycina*. The *C. almaatensis* extracts APCa and CECa, as well as afzelin, quercitrin and hyperoside isolated from the APCa have not shown the same peripheral anti-inflammatory effect in the present study when administered intraperitoneally at a concentration of 100 mg/Kg for extracts and 3mg/Kg for the isolated compounds. Perhaps, the lack of effect could be due to the administration of the extracts/compounds in very small doses.

To exclude a possible sedative effect masking the negative analgesic results, spontaneous activity tests in vivo were performed, namely open field and rotarod tests, using bigger doses of the extracts CECa and APCa. The absence in locomotor impairment when 100 mg/Kg of the APCa and CECa extracts were administered intraperitoneally is in agreement to the results obtained by Furtado et al. [35] using the extract of *Copaifera langsdorff* at the doses of 30, 100, 300 and 1000 mg/Kg as well as the major isolated compounds afzelin and quercitrin at the doses of 3, 10, 30 and 100 mg/Kg in the open field test. This excludes in the present work, a possible masking sedative effect on the nociceptive results.

Regarding the anti-inflammatory effect, APCa extract at a dose of 100 mg/Kg has shown inhibition of IL-1β and total leukocyte counts as mentioned before and the results are in agreement with Wang, Xiong and Feng [32] in which the aqueous extract of *Crataegus spp*. has demonstrated inhibition of COX-2, TNF-α e IL-1β and IL6 using an in vitro inflammatory model induced by lipopolysaccharide (LPS) [32]. In another study using a rheumatoid arthritis model of inflammation induced by LPS in vitro, hyperoside has demonstrated a significant (concentration-dependent) effect on inhibition of TNF-α, IL6 and IL-1β. The anti-inflammatory effect was attributed to a possible suppression in the activation of the NF-kβ pathway, which regulates the expression of several inflammatory mediators, including cytokines such as TNF-α, IL6, IL-1β, chemokines and cell adhesion molecules [37]. Hyperoside also has the ability to inhibit inflammasome activation [38] and Nrf2 [39], which could collective explain its anti-inflammatory profile. *Portulaca olearacea* [40] and *Protium spruceanum* [41], both rich in quercitrin, proved to suppress NF-kB/activate MAPK and exerted immunomodulation/enzyme activation, which could lead to their anti-inflammatory effect. Finally, the anti-inflammatory action of *Myracrodruon urundeuva* and *Tetraclinis articulata* is correlated to the presence of afzelin [42,43]. The presence of these three compounds as the main constituents of *Crataegus almaatensis* explains in part the anti-inflammatory action obtained for APCa.

## 5. Conclusions

The present work yielded the three main flavonoids (hyperoside, quercitrin and afzelin) from the leaves of *Crataegus almaatensis* (CECa and APCa). The novelty of the chemical investigation here is that no flavonoid constituent has previously been identified in this species. Also, this study achieved the optimization of the flavonoid isolation technique, delivering a higher number of isolated compounds in a shorter period of time. Possibly, using different polar solvent systems, other minor substances proven to be effective in the treatment of pain and cardiovascular diseases, could be isolated from *C. almaatensis* in future studies. Also, the vascular relaxation effect and the anti-inflammatory activity obtained when APCa was tested separately was not observed when CECa was tested at the same doses. The results achieved in this study represent the base for future work on this species, in order to confirm the pharmacological activity and elucidate the mechanisms of action for the extracts of the plant.

## Figures and Tables

**Table 1:** Maximum effect and potency for noradrenaline-induced vascular contractile response in the presence of different concentrations of the extracts crude extract-CECa (CECa) and aqueous phase of the crude extract (APCa).

Extract	Concentration (mg/mL)	% Emax	pEC_50_ (-log[M])	*r/n*
CE*Ca*	Control	100.00 ± 0.00	5.42 ± 0.11	11/4
	0.014	175.45 ± 42.56 *	5.79 ± 0.42	4/4
	0.028	83.60 ± 23.10	5.67 ± 0.33	3/3
	0.14	219.52 ± 141.52 *	5.24 ± 0.61	4/4
AP*Ca*	Control	100.00 ± 0.00	5.30 ± 0.08	7/4
	0.014	125.65 ± 102.12	5.72 ± 0.89	2/2
	0.028	43.41 ± 11.59	5.42 ± 0.27	2/1
	0.14	130.21 ± 58.65	4.98 ± 0.48	3/3

*r*, number of rings; *n*, number of arteries; Results are presented in mean ± SEM (*p < 0.05).
